# Decolorization and partial mineralization of a polyazo dye by *Bacillus firmus* immobilized within tubular polymeric gel

**DOI:** 10.1007/s13205-011-0035-3

**Published:** 2011-11-05

**Authors:** Chimezie Jason Ogugbue, Norhashimah Morad, Thomas Sawidis, Nathaniel A. Oranusi

**Affiliations:** 1 School of Industrial Technology, Universiti Sains Malaysia, 11800 Penang, Malaysia; 2 School of Biology, Aristotle University of Thessaloniki, Thessaloniki, Macedonia Greece; 3 Department of Microbiology, University of Port Harcourt, Port Harcourt, Nigeria

**Keywords:** Decolorization, Mineralization, Azo dye, Tubular gel, *Bacillus firmus*

## Abstract

The degradation of C.I. Direct red 80, a polyazo dye, was investigated using *Bacillus firmus* immobilized by entrapment in tubular polymeric gel. This bacterial strain was able to completely decolorize 50 mg/L of C.I. Direct red 80 under anoxic conditions within 12 h and also degrade the reaction intermediates (aromatic amines) during the subsequent 12 h under aerobic conditions. The tubular gel harboring the immobilized cells consisted of anoxic and aerobic regions integrated in a single unit which was ideal for azo dye degradation studies. Results obtained show that effective dye decolorization (97.8%), chemical oxygen demand (COD) reduction (91.7%) and total aromatic amines removal were obtained in 15 h with the immobilized bacterial cell system whereas for the free cells, a hydraulic residence time of 24 h was required for an equivalent performance in a sequential anoxic and aerobic process. Repeated-batch experiments indicate the immobilized cells could decolorize C.I. Direct red 80 and reduce medium COD in five successive batch runs with enhanced activity obtained after each consecutive run, thus suggesting its stability and potential for repeated use in wastewater treatment. UV–visible spectrophotometry and HPLC analysis were used to confirm the partial mineralization of the dye. Data from this study could be used as a reference for the development of effective industrial scale biotechnological process for the removal of dyes and their metabolites in textile wastewater.

## Introduction

Azo dyes are synthetic colored compounds which consist of a diazotized amine coupled to an amine or a phenol, and characterized by the presence of one or more azo bonds (N=N). These dyes dominate the worldwide market of dyestuffs with a share of about 70% (Mishra et al. 2011), making them the largest group of synthetic colorants and the most common dyes released into the environment (Saratale et al. 2009a; Zhao and Hardin 2007). During dye processing, as much as 2–50% of dyestuffs applied during dyeing of different substrates, such as synthetic and natural textile fibers, plastics, leather, paper, mineral oils, waxes, foodstuffs and cosmetics are wasted with spent dye baths due to their imperfect fixing properties (Tezcanli-Güyer and Ince 2004). If the wasted dye baths are channeled to the rest of the plant’s wastewater streams for treatment by conventional biochemical operations, the azo dyes may go through the system without degradation and ultimately reach the receiving water with the potential to produce carcinogenic amines under anaerobic conditions (Eren and Ince 2010). In addition, the strong color of discharged dyes even at very small concentrations has a huge impact on the aquatic environment since it has negative ecotoxicological effects on fauna, affects esthetic merit and results in reduction in light penetration and photosynthesis, thus posing pollution problems (Aksu et al. 2007; Manu and Chaudhari 2002). Therefore, adequate treatment of textile dyeing process effluents is imperative in order to remove these dyes and their reduction metabolites prior to their final discharge to the environment.

In the recent past, efforts have been made to develop efficient and sustainable methods for treatment of dye wastewater due to rigorous government legislations holding textile industries to increasingly higher standards of treatment regarding waste effluents (Corso and de Almeida 2009). Emerging technologies developed based on physical and chemical treatment methods for the removal of azo dyes from wastewater (Ai et al. 2010; Khataee et al. 2009; Li et al. 2007; Wang et al. 2009) were successful in some cases. However, these methods have not been widely applied because of their high cost, inability to completely remove the recalcitrant azo dyes and/or their organic metabolites and the secondary pollution that can be generated by the excessive use of chemicals (Forgacs et al. 2004; Zhang et al. 2004) Consequently, microbial degradation has been suggested as a viable alternative due to its eco-friendly nature and cost-competitiveness when compared to physicochemical treatment methods (Jadhav et al. 2010; Saratale et al. 2010).

The mechanism of bacterial degradation of azo dyes involves the reductive cleavage of azo bonds (–N=N–). This occurs with the help of azo reductase enzymes when carriers in the electron transport chain utilize azo compounds as terminal electron acceptors under anaerobic conditions which results in the formation of colorless solutions containing aromatic amines (Singh et al. 2007; Van der Zee and Villaverde 2005). This process is repressed by the presence of oxygen through its inhibition of the azo bond reduction activity since it is a more energetically favorable oxidizing agent. Hence, it is essential that bacterial decolorization is operated under nearly anaerobic conditions. However, aromatic amines resulting from the decolorization process in the anaerobic treatment are not mineralized under anaerobic conditions and tend to accumulate to toxic levels (Gottlieb et al. 2003; Luangdilok and Panswad 2000). Some of these aromatic amines are known to be cytotoxic, mutagenic and carcinogenic (Alves de Lima et al. 2007; Mansour et al. 2009) and would therefore pose a serious hazard if released into the aquatic environment untreated. Conventional processes for the removal of aromatic amines from industrial wastewaters include extraction, adsorption onto activated carbon, chemical oxidation, electrochemical techniques and irradiation. All of these methods suffer from drawbacks including high costs, formation of hazardous by-products and low efficiency (Gianfreda et al. 2003). Therefore, for the decomposition and removal of these putatively toxic and recalcitrant compounds, anaerobic biological treatment of dye wastewater will have to be followed by aerobic treatment (Libra et al. 2004). This observation has led to the proposal that anaerobic/aerobic systems might be effective in achieving the complete biodegradation of azo dyes.

In the last few years, several laboratory-scale sequential anaerobic/aerobic processes for the treatment of wastewater containing azo dyes have been described. Supaka et al. (2004) showed that the aerobic stage of combined anaerobic/aerobic treatment of dye wastes eliminated the chemical oxygen demand (COD), attributed to removal of the aromatic amines, which are anaerobically recalcitrant. The removal of aromatic amines was also evaluated during the decolorization of C.I. Direct black 38 dye using an anaerobic upflow anaerobic sludge blanket reactor (UASB) and an aerobic completely stirred tank reactor system (CSTR). Results obtained indicated that the total aromatic amines (TAAs) produced under anaerobic conditions were ultimately removed in the aerobic stage and aromatic amine recoveries such as 45–50% were obtained in the aerobic reactor (Sponza and Isik 2005). In another report, a sequential anaerobic/aerobic system was also used to treat a simulated textile wastewater containing azo dyes in which a significant part of TAAs was removed successfully in the sequential aerobic stage (Isik and Sponza 2008). Sequencing batch reactors (SBR) and other reactor types such as fluidized bed and packed bed reactors have also been used in recent studies to achieve dye removal (Sponza and Isik 2002; Ong et al. 2005). These presently available systems for dye degradation consists of two biological conversion steps (aerobic and anaerobic degradation), and require a large space for the aerobic and anaerobic units along with complicated operations. If the aerobic and anaerobic systems occurred in the same unit, dye removal systems would be simplified.

In this study, we discuss the partial mineralization of C.I. Direct red 80, a polyazo dye, in a single unit and compact aerobic and anoxic bioreactor system with simple operation and consisting of immobilized bacteria in tubular polymeric gel. The tubular gel has two regions: one region (near outer surfaces) of the tube contacts aerobically with the medium whereas, the other region (within tubular gel) remains anoxic. Azo dye partial mineralization in this integrated system occurred through the reductive cleavage of azo bonds in the anoxic region and the subsequent degradation of the resulting aromatic amines in the aerobic region of the tubular gel.

## Materials and methods

### Chemicals

**Fig. 1:**
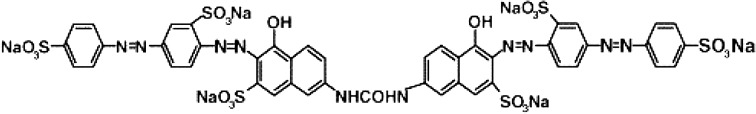
Chemical structure of C.I. Direct red 80 (C.I. 35780)

The polyazo dye (C.I. Direct red 80) used in this study was purchased from Huntsman Co. (formerly Ciba-Geigy) and was used as obtained. Figure 1 shows the chemical structure of the dye. The stock solution of the dye was prepared by membrane filtration. All other chemicals were obtained from Sigma Chemical Co. (St. Louis, MO, USA) and were of analytical grade.

### Microorganism and culture media

*Bacillus firmus* was isolated from dye wastewater collected from a textile industry as previously described (Ogugbue and Sawidis 2011). The bacterium was able to decolorize a variety of azo dyes under anoxic conditions via a pathway initiated by azo bond reduction (Ogugbue and Sawidis 2011).

Two media were used in this study: one was nutrient broth (NB) (Merck) used for routine transfer and cell cultivation, and the other was the synthetic wastewater medium (SWM) amended with C.I. Direct red 80. The basic composition of the synthetic dye wastewater was (g/L): (NH_4_)_2_SO_4_ 0.28, NH_4_Cl 0.23, KH_2_PO_4_ 0.067, MgSO_4_·7H_2_O 0.04, CaCl_2_·2H_2_O 0.022, FeCl_3_·6H_2_O 0.005, NaCl 0.15, NaHCO_3_ 1.0, yeast extract 0.2, dye 0.05 and 1 mL/L of a trace element solution containing (g/L): ZnSO_4_·7H_2_O 0.01, MnCl_2_·4H_2_O 0.1, CuSO_4_·5H_2_O 0.392, CoCl_2_·6H_2_O 0.248, NaB_4_O_7_·10H_2_O 0.177, and NiCl_2_·6H_2_O 0.02. Medium pH was adjusted to 7.2 with 1 M HCl or NaOH.

### Culture conditions for degradation

Batch decolorization assays were carried out with early-stationary phase culture of *B. firmus* in 250-mL Erlenmeyer flasks containing 100 mL of sterile SWM. The SWM in each flask supplemented with 50 mg/L of C.I. Direct red 80 was inoculated with 5% (v/v) freshly prepared inoculum of *B. firmus* and subsequently incubated at 30 °C for 15 h under aerobic (aeration at 100 mL/min) or anoxic (static incubation) conditions. Anoxic condition was progressively created and maintained in flasks by respiring bacterial cultures incubated under static condition. The same cell concentration of the isolate, adjusted at optical density 1.0 at λ = 620 nm (equivalent to ca. 4.87 logCFU/mL) was used in all experiments. The dye was filter-sterilized on a 0.45-μm filter (Millipore, USA) prior to addition to the sterile culture medium. During incubation, samples (5 mL) were withdrawn at 3-h intervals for determination of absorbance and percentage dye decolorization. Controls consisting of uninoculated flasks were also set up to determine the effect of abiotic factors on decolorization of C.I. Direct red 80.

To determine the fate of aromatic amines generated during biodegradation of azo dyes, batch sequential anoxic/aerobic culture experiments were carried using the SWM supplemented with 50 mg/L of C.I. Direct red 80. The experiment was started by inoculating the medium with the bacterium and incubating at 30 °C for 12 h under anoxic conditions or until no color was observed. Subsequently, same flasks were incubated under aerobic conditions as previously described for another 12 h at 30 °C. Abiotic control flasks were also set up and kept under similar conditions as the inoculated ones. At pre-determined intervals (every 3 h), samples were withdrawn from each flask for the determination of percentage dye decolorization, percentage COD reduction and residual TAA concentration.

### Cell immobilization

*Bacillus firmus* was grown in nutrient broth (Merck) in an orbital shaker (120 rpm) at 30 °C. The cells were harvested by centrifugation (3,824*g*, 10 min, 10 °C) and washed three times with physiological solution (0.85% NaCl). After washing, immobilization by entrapment using alginate polymer was carried out according to the method of Urkut et al. (2007). Freshly prepared cell suspension was mixed with an equal volume (1:1, v/v) of 4% (w/v) sodium alginate (Sigma, A-2033) solution with continuous stirring. The mixture was then transferred instantaneously to a mesh basket inside a mold containing sterile 0.2 M CaCl_2_ to initiate polymerization and the gel formed was allowed to harden for 30 min. The tubular polymeric gel (ca. 26.5 cm^3^) was washed with sterile physiological solution to remove excess calcium ions and cells and then incubated overnight at 28 °C to stabilize the polymer. A glass tube was used as the mold to form the polymeric gel containing the bacterial cells into a tube (outside diameter 22 mm, inside diameter 8 mm, and length 80 mm). The tubular gel was washed severally in sterile phosphate buffer before use.

### Batch mineralization experiments using free and immobilized cells

**Fig. 2:**
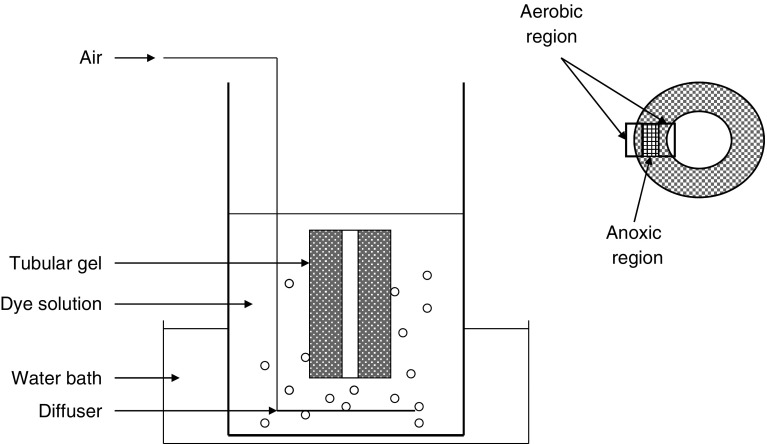
Schematic diagram of the batch-operated bioreactor system used in this study showing the tubular polymeric gel inside (*inset* cross-section of tubular gel showing anoxic and aerobic regions)

The synthetic wastewater (250 mL) containing C.I. Direct red 80 (50 mg/L) was treated using the tubular polymeric gel system (Fig. 2) at 30 °C with aeration (100 mL/min) in a bioreactor. The reactor was made of Plexiglas with a reaction liquid volume of 400 mL. Decolorization assay using free cells was also carried out using a similar bioreactor in which 250 mL of SWM supplemented with 50 mg/L C.I. Direct red 80 was used. Dye decolorization, COD reduction and TAA removal were monitored with time in both reactors placed under the same conditions. However, experiments with immobilized cell system were operated batch-wise for 12 h while assays with free cells consisted of anoxic (12 h) and aerobic (12 h) phases for a total hydraulic residence time of 24 h. The reaction temperature was controlled at 30 °C using a water bath. At intervals (every 3 h), samples were withdrawn from bioreactors and centrifuged at 15,000*g* for 15 min. A slight mixing was provided during the anoxic phase in free-cell assay to obtain homogenous conditions before sample collection. The absorbance of the supernatants was determined using a spectrophotometer to check for color removal. The supernatants were also analyzed for residual COD and TAA in order to evaluate the degradation of aromatic amines and ultimately, dye mineralization.

### Repeated-batch operations of decolorization with immobilized cells

Repeated-batch operations were performed with the immobilized cells to justify repetitive use. The performance of the tubular polymeric gel bioreactor was evaluated by following the decolorization extents for consecutive five batch runs, with a renewal of the decolorization medium carried out every 12 h. The bioreactors were operated under previously described conditions and each batch run consisted of anoxic/aerobic reaction (12 h), sample collection period (20 min) and fill and set up (40 min). During the fill and set-up operation, the polymeric gel was retrieved, rinsed thrice with sterile physiological saline, and transferred into a fresh decolorization medium for the subsequent decolorization experiment. Withdrawn samples were used for the determination of % dye decolorization and % COD reduction after each batch run.

### Analytical methods

Color removal was monitored by analysis of absorbance of withdrawn samples using a scanning spectrophotometer (UV/visible, Shimadzu, Kyoto, Japan) against a baseline defined by the absorbance of clarified samples from dye free SWM. The absorbance of each clarified supernatant was measured at the maximum absorption wavelength (λ_max_) of the dye (545 nm). The residual dye concentration in samples was determined from the absorbance readings using the calibration curve for absorbance versus dye concentration obtained by plotting the corresponding maximum absorbance in the UV–visible spectra at different concentrations of dye.

The % decolorization (*D*%) was calculated using the following equation:where *C*_0_ and *C*_1_ represent the initial and residual concentrations of azo dye, respectively. Average values of replicates were used in calculations. TAA was measured using a colorimetric method in a spectrophotometer at 440 nm after reacting with 4-dimethylamino benzaldehyde–HCl (Oren et al. 1991). Measurement of soluble COD was performed according to the procedure described in Standard Methods for the Examination of Water and Wastewater (APHA 1998). The % COD reduction (CR%) was calculated as follows:where COD_0_ and COD_t_ are the initial COD value (at 0 h) and the observed COD value after a particular reaction time (*t*), respectively. All experiments were carried out in duplicates. Data obtained were subjected to statistical analysis to determine means and standard deviations of means. Significant differences between means of experiments were determined by one-way analysis of variance (ANOVA) with Tukey–Kramer multiple comparisons test. Readings were considered significant when *P* value was ≤0.05.

### HPLC analysis

HPLC analysis was carried out to follow the biodegradation of the aromatic amines during bioreactor operation by the immobilized cells. Analysis was done using a Shimadzu Liquid model LC-10AD chromatograph (Shimadzu Corp., Kyoto, Japan) equipped with Shimadzu model SPD-MIOAVP photodiode array detector and Inertsil ODS (column with 4*.*0 mm × 250 mm inside diameter, 5 μm, MZ Analysentechnik, Mainz, Germany). A mobile phase, consisting of 70% methanol and 30% water, was pretreated with helium to remove residual gases in solution, before it was fed into the HPLC at a flow rate of 1 mL/min. The eluates were monitored by UV absorption at 240 nm.

### Scanning electron microscopy (SEM)

In order to verify bacterial entrapment in polymeric gel matrix, micrographs were taken by SEM. The sliced gel after fixation and dehydration was critical point dried in a Balzers CPD 030 device and then coated with carbon in a JEE-4X vacuum evaporator. Observations were made with a JSM 840-A scanning electron microscope and images were captured digitally.

## Results and discussion

**Fig. 3:**
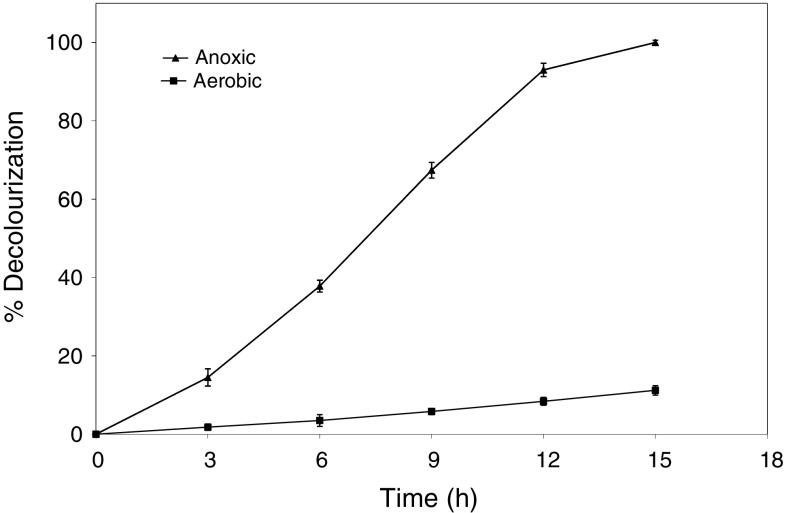
Decolourization extent obtained during degradation of C.I. Direct red 80 by *Bacillus firmus* under anoxic and aerobic conditions

Preliminary decolorization experiments using free cells were carried out to determine the ability of *B. firmus* to decolorize C.I. Direct red 80 under aerobic and anoxic conditions. Results obtained show that extensive decolorization of the dye by the bacterial isolate was obtained when decolorization assay was performed under anoxic conditions while incubation under aerobiosis resulted in poor decolorizaton of dye (Fig. 3). At 12 h of incubation under anoxic conditions, the dye decolorization extent was 93% while complete decolorization of the dye was obtained after 15 h. However, under aerobic conditions, only 11.2% decolorization of the dye was obtained after 15 h of incubation. This clearly indicates that the decolorization of the polyazo dye by *B. firmus* was more efficient under anoxic conditions rather than under aerobiosis. The first step in the bacterial degradation of azo dyes is the reduction of the –N=N– bond which results in decolorization. This reaction is usually catalyzed by the oxygen sensitive enzyme, azoreductase, which is produced constitutively in dye degrading bacterial strains (Pandey et al. 2007). Hence, the effective decolorization of C.I. Direct red 80 in this study under anoxic conditions may be attributed to the cleavage of the chromophoric azo bonds of the polyazo dye by the azoreductase of *B. firmus.* The structural makeup of C.I. Direct red 80 may have contributed to its poor biodegradation under aerobiosis as azo dyes containing mainly nitro and sulfonic groups have been reported to be recalcitrant to aerobic bacterial degradation (Claus et al. 2002). This fact is probably related to the electron-withdrawing nature of the azo bond and their resistance to oxygenases attack. As anaerobic color removal occurs by the way of reduction of the azo dye, which acts a final electron acceptor in the microbial electron transport chain, existing and more effective electron acceptors like oxygen in the medium could act as a limiting factor for dye removal (Çinar and Demiröz 2010). Similar results were obtained in studies on pure bacterial strains such as *Pseudomonas luteola, Pseudomonas* sp. *SUK1* and *Micrococcus glutamicus NCIM*-*216*8 (Chang et al. 2001; Kalyani et al. 2008; Saratale et al. 2009b). The biodegradation of the azo dye C.I. Acid orange 7 by *Pseudomonas* sp. OX1 was studied under a variety of operating conditions and it was found that the decolorization potential of the microorganism was fully exploited only in the absence of oxygen (Lodato et al. 2007). In another study, Forgacs et al. (2004) also observed that the efficiency of aerobic treatment is inferior to that of anaerobic decolorization process. These earlier reports suggested that aeration and agitation, which increases the concentration of oxygen in the solution, should be avoided for efficient color removal. Nevertheless, the intermediate products (carcinogenic aromatic amines) generated from anaerobic breakdown of azo dyes have to be degraded by an aerobic process (Forgacs et al. 2004).

**Fig. 4:**
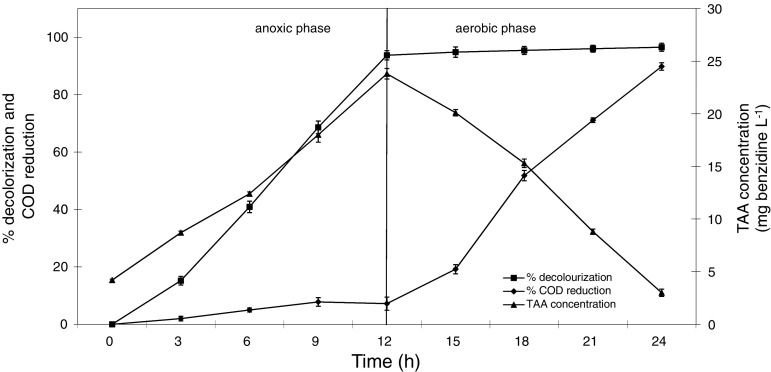
Phase-curve profiles of % decolourization, % COD reduction and residual TAA concentration during degradation of C.I. Direct red 80 by free cells of *Bacillus firmus* under sequential anoxic–aerobic conditions

In another experiment carried out to determine the fate of aromatic amines generated during the break down of the polyazo dye, results obtained show that the aromatic amines were persistent under anaerobic conditions even after prolonged incubation (data not shown) and were only degraded by the bacterial cells when they were kept under aerobic conditions in a two-cycle operation during a sequential anoxic–aerobic biodegradation tests. Filtrates obtained using a membrane filter (0.45-μm pore size) after anoxic incubation and kept under aerobic conditions did not show reduction in TAA suggesting that the aromatic amines were not autoxidized in the presence of oxygen. As presented in Fig. 4, TAA levels increased from 4.2 to 23.8 mg/L during the anoxic process (first 12 h) while a slow reduction in COD (7.2%) was obtained during same process. However, the onset of aerobiosis (12–24 h) resulted in a significant reduction in TAA (from 23.8 to 3.0 mg/L) and a marked reduction in COD to a total of 89.8% from 7.2% initially obtained during the anoxic phase, thus indicating the preference of an aerobic environment by the bacterium for the breakdown of the generated aromatic amine intermediates. Similarly, Isik and Sponza (2008) found that azo dyes (C.I. Reactive black 5, C.I. Direct red 28, C.I. Direct black 38, C.I. Direct brown 2 and C.I. Direct yellow 12) could be decolorized under reductive anaerobic conditions in very short hydraulic residence times but the breakdown products were not ultimately metabolized and accumulated under anaerobic conditions. However, in the sequential aerobic stage, the significant part of TAA was removed successfully with removal efficiencies of 70–85% obtained at total hydraulic residence times of 8.85 days. Tan et al. (2000) found that C.I. Mordant yellow 10 was reductively cleaved and the resulting aromatic amines, 5-aminosalicylic acid and sulphanilic acid, were both recovered under anaerobic conditions. These aromatic amines were ultimately mineralized under aerobic conditions. In another study performed by Isik and Sponza (2003), it was reported that an anaerobic (upflow anaerobic sludge blanket reactor)/aerobic (completely stirred tank reactor) sequential reactor system was used to remove 88% COD, 99% color and 91% TAA in synthetic wastewater containing Congo red dye. The TAA produced under anaerobic conditions was ultimately removed in the aerobic stage, resulting in very low aromatic amine recoveries (5–18%) in the last one. It had also been previously reported that aromatic amines can be mineralized by means of aerobic treatment by non-specific enzymes via hydroxylation and ring-opening in the presence of oxygen (Chang et al. 2001; Pandey et al. 2007). Usually, the reductive cleavage of the azo bond dissipates the electron deficiency of the aromatic nuclei so that the aromatic amino compounds generated may be subject to subsequent oxidation and mineralization (Forgacs et al. 2004). Hence, previous reports have suggested using an anaerobic process with a subsequent aerobic treatment for the removal of azo dyes in wastewater and to improve the biodegradability of generated aromatic amines prior to their final discharge to the environment (Khehra et al. 2006; You and Teng 2009). The results of this study further corroborate the fact that the breakdown of aromatic amines generated from azo dyes occurs via biodegradation under aerobic conditions.

**Fig. 5:**
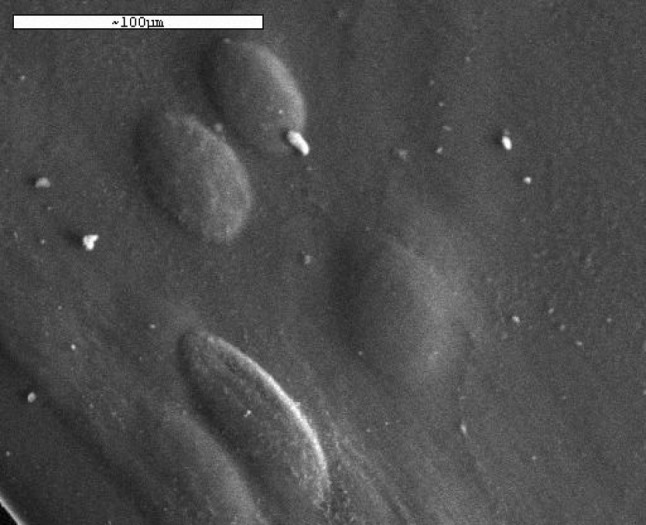
SEM photomicrograph of cells of *Bacillus firmus* entrapped within Ca-alginate matrix of the tubular gel

Consequently, an integrated system incorporating both anoxic and aerobic environments within one system for C.I. Direct red 80 decolorization and the degradation of the resulting aromatic amines by *B. firmus* was designed in the form of a tubular polymeric gel with immobilized bacteria (Fig. 2). The bacterial cells were entrapped and retained within the gel where they accessed the dye and its degradation intermediates through diffusion. The SEM of the cut tubular gel presented in Fig. 5 shows the bacterial cells homogenously dispersed and embedded within the alginate gel. Alginate immobilization is the most commonly used bacterial gel entrapment method for many bioremediation applications (Raja Noor et al. 2006) and offers several advantages such as protection of bacterial cells against environmental conditions, enhancement of activity in most cases and ensuring good diffusion into the polymer (Galai et al. 2010).

**Fig. 6:**
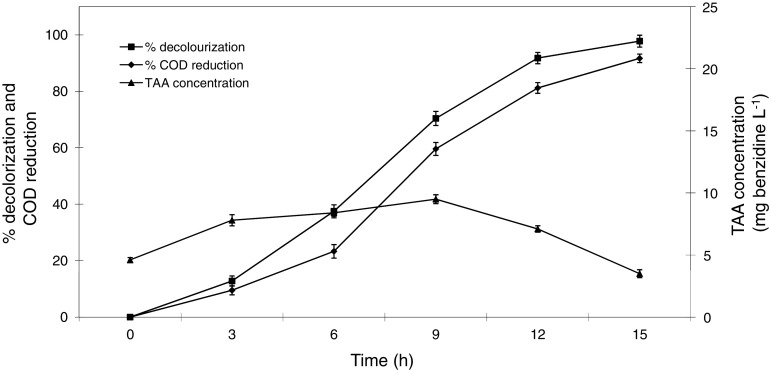
Profiles of % decolourization, % COD reduction and residual TAA concentration during degradation of C.I. Direct red 80 by tubular gel-immobilized cells of *Bacillus firmus* under aerobic conditions

**Fig. 7:**
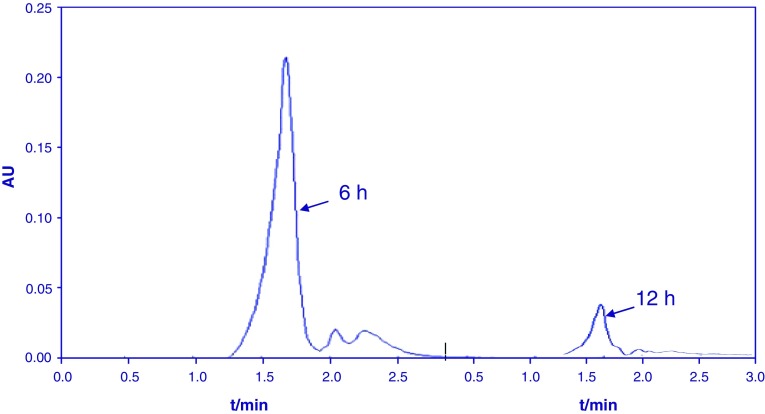
HPLC chromatograms of medium obtained from the immobilized cell system after 6 and 12 h hydraulic residence times

**Fig. 8:**
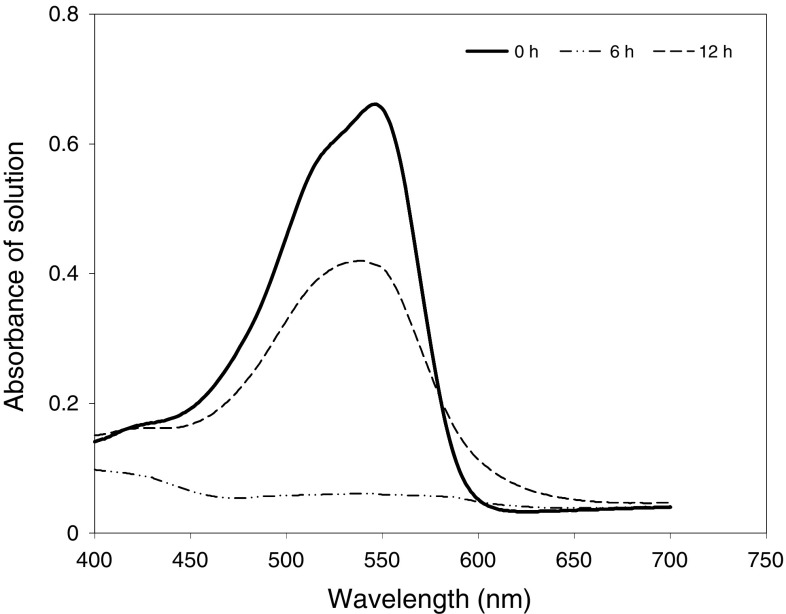
UV–visible spectra of C.I. Direct red 80 before (0 h), during (6 h) and after (12 h) decolorization by immobilized cells of *Bacillus firmus*

Data obtained when the immobilized cell system was used for degradation studies are as presented in Fig. 6. Results indicate almost complete decolorization of C.I. Direct red 80 was obtained within a hydraulic residence time of 12 h with the immobilized cells. The decolorization extent obtained with the immobilized cell system was 91.8% at an aerobic hydraulic residence time of 12 h, whereas 93.7% was obtained with the free cells at same hydraulic residence time under anoxic conditions. The slight difference in decolorization extents obtained for both systems at 12 h could be attributed to mass transfer diffusion limitation in the polymeric gel. On the other hand, changes in TAA and % COD reduction with time show marked deviations in trends when compared to data obtained during the sequential anoxic–aerobic degradation experiments with free cells (Fig. 4). Increase in TAA was from 4.6 to 9.5 mg/L in 9 h followed by a decrease to 3.5 mg/L after a total aerobic hydraulic residence time of 15 h in the immobilized cell system. Likewise, COD removal was more rapid with immobilized bacteria in tubular polymeric gel than when free cells were used in the sequential anoxic–aerobic system. With the immobilized cell system, COD reduction was from 0 to 91.7% at a total aerobic hydraulic residence time of 15 h, whereas with the free cells, % COD reduction was from 0 to 89.8 at a total hydraulic residence time of 24 h with higher COD reduction (82.6%) occurring during the 12-h aerobic incubation phase. Thus, higher COD and TAA removal were obtained with immobilized cells of *B. firmus* when compared to the free cells. The existence of anoxic and aerobic regions within the tubular polymeric gel may have been instrumental to the rapid degradation of C.I. Direct red 80 and its resulting aromatic amines by the immobilized bacterial cells, thus leading to COD removal and partial mineralization of the dye. Owing to the low solubility of oxygen in water and the high local cell density, oxygen transfer often becomes limiting and results in the creation of an anoxic center. The dye was probably decolorized at the anoxic center of the gel while the aromatic amines were degraded at the outer aerobic zones of the gel (Fig. 2), thus presenting the use of immobilized bacteria in tubular polymeric gel as an effective method of achieving both anoxic and aerobic environments in one integrated process. The drop in % COD reduction in the free-cell system between anoxic hydraulic residence times of 9 and 12 h may have been due to the accumulation of small organic compounds (metabolites of azo bond cleavage) that are recalcitrant to anoxic degradation but easily amenable to oxidative degradation, thus resulting in an increased COD value. HPLC chromatographs obtained during the course of the experiment with the immobilized cell system show a decrease in aromatic amine peak obtained with time suggesting partial mineralization of C.I. Direct red 80 (Fig. 7). At 6-h hydraulic residence time, an intermediate metabolite (retention time 1.6 min) accumulated accompanying the increasing decolorization of C.I. Direct red 80. This intermediate metabolite decreased significantly after 12-h hydraulic residence time without the emergence of alternate major peaks in the decolorized medium. However, the chromatographic peaks could not be identified due to unavailability of authentic standards. These results suggest that the azo dye structural bone was degraded. Figure 8 shows the UV–visible spectral scans obtained at different times (0, 6, 12 h) during the decolorization of C.I. Direct red 80 by the immobilized cell system. The absorbance signature of the azo dye consisted of a major peak in the visible region (at 545 nm) within the range of wavelength scanned (400–700 nm). The absorbance spectrum reduced progressively with time and there was no formation of new peaks. A reduction in absorbance in the visible spectrum indicates dye decolorization through cleavage of the chromophoric group (–N=N–). In addition to decolorization by immobilized cells, adsorption of dye molecules to the gel matrix may also play a role in decolorization experiments. Cell-free tubular polymeric gels were prepared and examined for their non-enzymatic color removal ability using an initial dye concentration of 50 mg/L and results indicate abiotic decolorization extent of less than 5% at equilibrium (data not shown). Hence, physical adsorption of dye to the polymeric gel was neglected when calculating decolorization extent of the immobilized cells.

The ability to separate anoxic and aerobic regions horizontally across the tubular gel is probably the greatest advantage of this type of integrated system as it allows the matrix to behave as a two-phase system without the associated control problems and high cost of sequential anaerobic–aerobic systems. A similar artificial ecosystem system was used in nitrogen removal in wastewater in which aerobic and anaerobic zones were provided simultaneously within the gel used for nitrification and denitrification processes (Uemoto and Saiki 1996). The use of immobilized microbial cells has received increasing interest in the field of wastewater treatment. For example, Su et al. (2009) used immobilization of anthraquinone and quinine-reducing bacterial consortium via entrapment in calcium alginate to accelerate azo dye biodecolorization. Chen and Lin (2007) entrapped a Gram-negative bacterium *Pseudomonas luteola* in beads for azo dye decolorization. Couto et al. (2004) used polyurethane foam (PUF), nylon sponge, alginate beads as carrier materials to immobilize white-rot fungus for textile dye decolorization. Immobilized cell systems have the potential to degrade toxic chemicals faster than conventional wastewater treatment systems since high densities of specialized microorganisms are used (He et al. 2004). In this study, results show that the tubular gel bioreactor can effectively degrade C.I. Direct red 80 through an integrated anoxic–aerobic process and this is the first demonstration of azo dye partial mineralization using bacterial cells immobilized in a tubular polymeric gel.

**Fig. 9:**
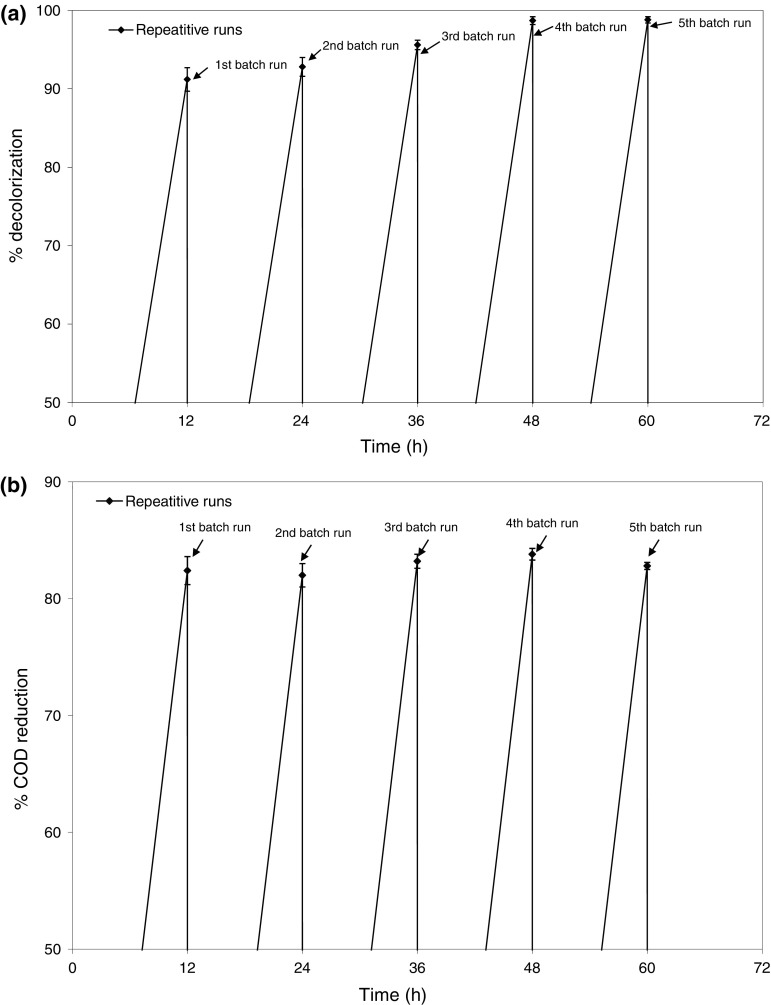
Fed-batch decolorization extents (**a**) and % COD reduction (**b**) obtained during repetitive runs carried out using the immobilized cell system. Each batch run was for 12 h

Repeated-batch experiments performed with the immobilized cell system indicated its potentials for reusability. Five consecutive batch runs were carried out and the decolorization extent obtained increased with each successive batch run up to the fifth run (Fig. 9). The % decolorization of C.I. Direct red 80 after the first batch run was 91.2, while decolorization extents obtained after the third and fifth batch runs were 95.6 and 98.8%, respectively. Reduction in COD after each successive batch operation was stable through the five runs. This indicates that the tubular polymeric gel offered stability for bacterial activity and enhanced decolorization upon acclimation due to adaptation effect. However, decrease in decolorization extent and % COD reduction was obtained in successive batch runs beyond the fifth probably due to bulging of the gel and mechanical instability (data not shown). The application of the tubular polymeric gel shows good potentials for separation and recovery of the immobilized bacteria, simplified operation, cost effectiveness and reusability. Suspended cultures are not technically viable for long-term repetitive batch operations due to total washout of microbial cells during operation. Reasons for reusability of immobilized cell systems have been deduced by many authors. Roukas and Kotzekidou (1991) worked on lactic acid production using immobilized cells and attributed this ability of immobilized cells to produce lactic acid for a long time to the protection of cells by the immobilization matrix. In another report, the enhanced performance of immobilized cells as compared to free cells and their ability to retain metabolic activities for a long time were attributed to the maintenance of high cell density without washout in bioreactors, the tendency for higher activity, and the tolerance of bound-cell systems to perturbations in the reaction environment and to toxic substances present in the liquid medium (Couto 2009). High cell densities within the immobilization matrix and stabilization of activity of strains through prevention of plasmid loss were also advanced as reasons for sustained performance of immobilized cell systems (Cachon and Divies 1993). In this study, the good polymerization conditions of the calcium alginate gel and the direct role that the calcium plays in cell conservation (Tamponnet et al. 1989) may have also played a role. During the repeated-batch experiments, slight cell leakage from the tubular gel was observed especially after the third batch run. However, the leakage of cells from the gel did not affect the efficiency of the degradation process or the integrity of the tubular gel up until the fifth run. Other researchers have also reported cell leakage while using Ca-alginate beads (Goksungur and Guvenc 1999; West and Strohfus 2001), and many attempts have been made to decrease cell leakage and to improve the stability of Ca-alginate beads through coating of beads with chitosan (Goksungur et al. 2005) or treating the beads with poly-l-lysine, polyethyleneimine, glutaraldehyde or hexamethylenediamine (Bodalo et al. 1997). It was stated that some of those agents prevented leakage but also caused drastic losses in cell viability. Thus, enhancing the stability of Ca-alginate tubular gel, prevention of cell leakage and the identification of alternative immobilization matrices with good mechanical strength and high rigidity to withstand the high shear condition usually encountered in a process stream could be subjects of further research. Furthermore, the performance of this simple but yet cost-effective technology on a larger scale needs to be evaluated.

## Conclusions

In this study, an efficient integrated anoxic/aerobic system for the partial mineralization of azo dyes by *B. firmus* immobilized within a tubular polymeric gel was investigated. The Ca-alginate tubular gel consisted of anoxic and aerobic regions which facilitated the decolorization of azo dyes in the anoxic center and the degradation of resulting aromatic amines from azo bond cleavage at the aerobic region. Data obtained during batch experiments indicated that the tubular polymeric gel was efficient in dye decolorization, COD reduction and TAA removal from medium supplemented with C.I. Direct red 80. Shorter hydraulic residence time (12 h) for color removal, COD reduction and TAA removal was obtained with the immobilized bacterial cell system when compared to the free cells which required a hydraulic residence time of 24 h for an equivalent performance. The adsorption of dye on the Ca-alginate tubular gel was shown to be insignificant, while control experiments showed dye decolorization, COD reduction and TAA removal were due to activities of bacterial cells. Repeated-batch operations were also performed with the immobilized cell system to justify reusability and results obtained indicate its stability and enhanced performance with each successive run on acclimation for five runs. The study data suggest that tubular polymeric gel, utilizing immobilized bacterial cells as biocatalyst, could be used as an integrated aerobic and anoxic system for the partial mineralization of azo dyes. This immobilized cell technology could serve as a reference for the development of bioreactors with an integrated anoxic/aerobic system for treatment of textile industrial wastewater since it offers minimal space requirement, low capital cost and good COD removal efficiency.
